# Study on Modulation Bandwidth of GaN-Based Micro-Light-Emitting Diodes by Adjusting Quantum Well Structure

**DOI:** 10.3390/nano12213818

**Published:** 2022-10-28

**Authors:** Pan Yin, Ting Zhi, Tao Tao, Xiaoyan Liu

**Affiliations:** 1 Institute for Electric Light Sources, School of Information Science and Technology, Fudan University, Shanghai 200438, China; 2 College of Integrated Circuit Science and Engineering, and National and Local Joint Engineering Laboratory for RF Integration and Micro-Packaging Technologies, Nanjing University of Posts and Telecommunications, Nanjing 210023, China; 3 Key Laboratory of Advanced Photonic and Electronic Materials, School of Electronic Science and Engineering, Nanjing University, Nanjing 210046, China

**Keywords:** micro-light-emitting diodes, modulation bandwidth, quantum-confined Stark effect

## Abstract

GaN-based blue micro-light-emitting diodes (μ-LEDs) with different structures were designed, of which the effect of quantum well (QW) structure on modulation bandwidth was numerically explored. By using trapezoidal QWs, the quantum-confined Stark effect (QCSE) can be reduced, leading to an enhanced electron-hole wave function overlap, thereby increasing the recombination rate and reducing the differential carrier lifetime. In addition, the improved hole transport also creates favorable conditions for shortening the differential carrier lifetime. Furthermore, by comparing with traditional μ-LEDs with different thicknesses of QW, the modulation bandwidth of μ-LEDs with trapezoidal QWs exhibits a large advantage at lower current densities of below 2 kA/cm^2^.

## 1. Introduction

Light-emitting diode (LED) has undergone great developments since its birth, penetrating every corner of our lives. Recently, great opportunities for LEDs are beginning to emerge in visible light communications (VLC) [1,2]. However, the major factor limiting the performance of GaN/InGaN LED-based VLC systems is the modulation bandwidth of the LEDs, which is far from the needs of modern wireless communication systems, creating an enormous obstacle to its application in VLC [3,4].

The modulation bandwidth of LEDs is inversely related to the Resistance–Capacitance (RC) time constant and the carrier lifetime [5,6], and generally, the larger one plays a major role. The pixel size of LEDs has been reported to be an important factor affecting their inherent RC time constant [5,6,7]. Thus, the limitation of the RC time constant on the modulation bandwidth of LEDs can be reduced by reducing the size of the LEDs [8,9,10]. Previous studies have found that the modulation speed of LEDs can be increased by reducing the thickness of the quantum barrier (QB), due to the enhancement of the recombination rate caused by the amelioration of the carrier distribution [11]. Carrier lifetime is also a key factor of the frequency response performance and usually decreases with increasing current density [12,13]. Numerous studies have shown that high current density is the cornerstone for supporting high modulation speed [14,15,16]. With this background, excellent thermal performance, the negligible RC time constant [8,12], and high operating current density make micro-light-emitting diodes (μ-LEDs) one of the most attractive candidates for high-speed VLC applications [17,18,19,20,21]. However, a disadvantage in polar GaN/InGaN-based μ-LEDs is the quantum-confined Stark effect (QCSE), in which the polarization electric field separates the wave functions of electrons and holes, leading to a reduction in the recombination rate and an increase in carrier lifetime [22,23,24]. Besides, high operating current densities often outweigh the benefits, as the resulting efficiency droop, so it makes sense to achieve higher modulation bandwidths at lower current densities [25]. Compared with conventional μ-LEDs grown on the c-plane, semi-polar and non-polar μ-LEDs exhibit weaker QCSE, higher overlaps of electron-hole wave functions, and shorter carrier lifetimes, attracting more and more research interest in recent years [9,16,21,25,26].

Through the great efforts of researchers, the modulation bandwidth of μ-LEDs measured in the laboratory has been significantly improved [8,27,28,29]. However, most studies focused on the improvement of modulation bandwidth by adopting new materials and new LED structures, but the mechanisms have not been well analyzed, which is crucial to developing μ-LEDs with high modulation bandwidth. It is vital to study the influence of the effects of quantum well (QW) structures on the modulation bandwidth and to clarify the underlying physical mechanism, which will provide a valuable guide for μ-LEDs fabrication in future VLC applications.

Simulation provides a device design method without relying on the epitaxial process, saving development costs. In this article, APSYS (an acronym for Advanced Physical Models of Semiconductor Devices) has been used to study the modulation bandwidth of GaN-based blue μ-LEDs with different QW structures for VLC applications [30]. In addition, the effect of band structure has been taken into account, and a series of diagrams have been constructed to illustrate the physical mechanisms involved.

## 2. Theoretical Model and Device Structure

The carrier recombination rate (*R*) is defined as the number of carriers recombined per unit time and unit volume. Based on the ABC efficiency model [31,32,33], the *R* is mainly composed of Shockley–Read–Hall (SRH) recombination, radiative recombination, and Auger recombination, which are proportional to the first, second, and third power of the carrier concentration, respectively [34,35,36]. The carrier generation rate (*G*) is defined as the number of carriers generated per unit time and unit volume. Under equilibrium conditions, the carrier generation rate in the active region of a μ-LED is approximately equal to the recombination rate in it, expressed by the following formula [35,37]:

(1) $$G=R=An+Bnp+C\left(n^{2}p+p^{2}n\right)$$

where *A*, *B*, and *C* represent SRH recombination coefficient, radiative recombination coefficient, and Auger recombination coefficient, respectively, *n* represents electron concentration, and *p* represents hole concentration.

Under high current density, the excess carriers dominate, while the excess electron concentration balances with the excess hole concentration [24,38,39,40,41]. When a high-frequency small-amplitude signal is injected at high current density, the increase in electron concentration is equal to that of hole concentration, and the increase in carrier concentration is much smaller than the carrier concentration at direct current (DC) bias. Therefore, the relationship between the increment of the carrier generation rate (Δ*G*) and the increment of the carrier concentration is (Δ*n*) as follows:

(2) $$\begin{array}{cc} \Delta G= & A\left(n+\Delta n\right)+B\left(n+\Delta n\right)\left(p+\Delta n\right)\\ & +C\left({\left(n+\Delta n\right)}^{2}\left(p+\Delta n\right)+\left(n+\Delta n\right){\left(p+\Delta n\right)}^{2}\right)\\ & -\left(An+Bnp+C\left(n^{2}p+np^{2}\right)\right)\\ \approx & A\Delta n+B\left(n+p\right)\Delta n+C\left(n^{2}+p^{2}+4np\right)\Delta n \end{array}$$

The differential carrier lifetime (*τ*) can be obtained from the derivative of the carrier generation rate with respect to the carrier concentration, expressed by the following formula [12,42]:

(3) $$\frac{1}{\tau }=\frac{\Delta G}{\Delta n}=A+B\left(n+p\right)+C\left(n^{2}+p^{2}+4np\right)$$

In the frequency response of μ-LEDs, the −3 dB modulation bandwidth (*f_−3dB_*) is defined as the corresponding frequency when the normalized power drops to half of the maximum value. Generally, the differential carrier lifetime has a relationship with the 3 dB modulation bandwidth of the LED as follows [12,25]:

(4) $$f_{-3dB}=\frac{1}{2\pi \tau }$$

In the physical model of simulation, band offset, internal loss, and the SRH recombination lifetimes are set to 70:30, 2000 m^−1,^ and 200 ns [34,43,44], respectively. Moreover, the Auger recombination coefficient is set to 3 × 10^−31^ cm^−6^/s [43,44]. Built-in polarizations ranging from 20% to 80% of theoretical predictions have been reported, and 50% are chosen for simulation in this study [34,45,46]. Other physical parameters can be found in references [47].

**Figure 1:**
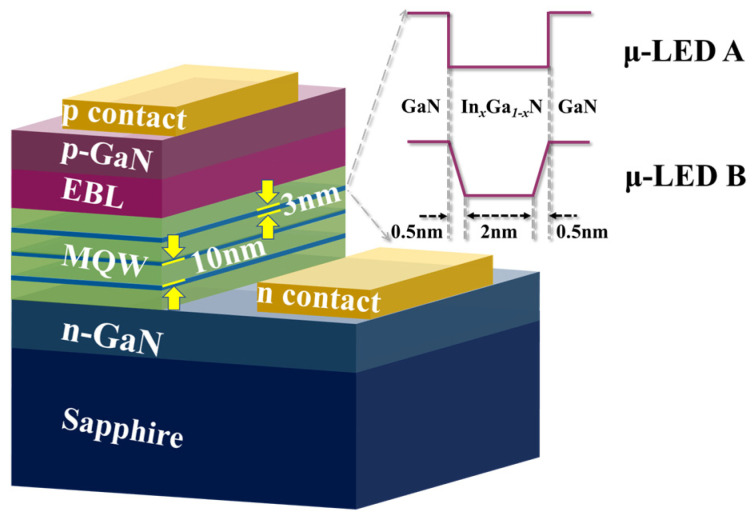
μ-LEDs with different structures for simulation: μ-LED A with conventional QWs and μ-LED B with trapezoidal QWs.

The structures of the μ-LEDs in this work are shown in Figure 1. There is a layer of 10 μm thick sapphire substrate at the bottom, followed by a 3 μm thick GaN layer with an n-type doping concentration of 5 × 10^18^ cm^−3^ and a three-period GaN/InGaN multiple quantum well (MQW) layer. The thickness of QBs is 10 nm, where the n-type doping concentration is 3 × 10^17^ cm^−3^. The indium content in QWs is set to 20% to ensure an emission at a wavelength of around 450 nm. On the top of the active area, there is a 20 nm thick Al_0.23_Ga_0.77_N as an electron blocking layer (EBL) with a p-type doping concentration of 1.2 × 10^18^ cm^−3^ and a 50 nm thick GaN as a cladding layer with a p-type doping concentration of 1.2 × 10^18^ cm^−3^. The ohmic contact on the cladding layer is defined as the p-electrode of the μ-LED and that on the n-type GaN layer is defined as the n-electrode of the μ-LED.

To study the effect of QWs on the modulation bandwidth, μ-LEDs with two different QW structures have been designed, represented by μ-LED A and μ-LED B in Figure 1. The size of the μ-LED is defined as 20 μm × 20 μm, making the influence of the RC time constant negligible. For μ-LED A, the thickness of one QW is 3 nm with an indium composition of 0.2. For μ-LED B, the thicknesses of the falling side, bottom, and rising side of one QW are 0.5 nm, 2 nm, and 0.5 nm, respectively, with an indium composition ranging from 0 to 0.2. This design enables the two μ-LEDs with the same QW thickness, supporting the subsequent comparative analysis.

## 3. Results and Discussion

**Figure 2:**
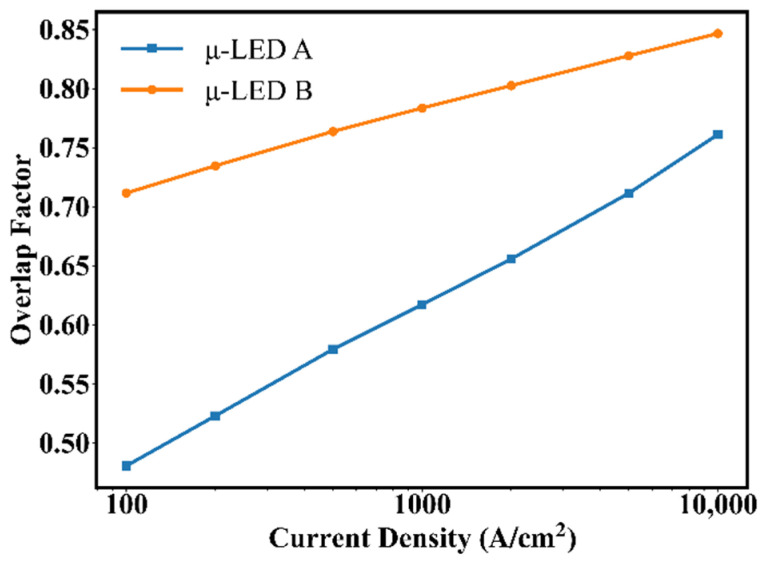
Overlaps of electron-hole wave function vs. current density for μ-LED A and μ-LED B.

Figure 2 shows the overlap of the electron-hole wave function as a function of current density for μ-LED A and μ-LED B. It can be found that the overlap is far below 1 at low current density due to the separation of the wave functions of electrons and holes caused by QCSE [48]. In addition, the overlaps of μ-LED B are higher than those of μ-LED A, owing to the trapezoidal QW, where there is less lattice mismatch and weaker QCSE, resulting in less separation of electrons and holes [49]. Furthermore, the overlap increases as the current density increases due to the band-filling effect that counteracts the separation of carriers, which also leads to a reduction in the gap between the overlaps of μ-LED A and μ-LED B [37,48]. The above demonstrates that the trapezoidal QW can improve the electron-hole wave function overlap and attenuate the QCSE.

**Figure 3:**
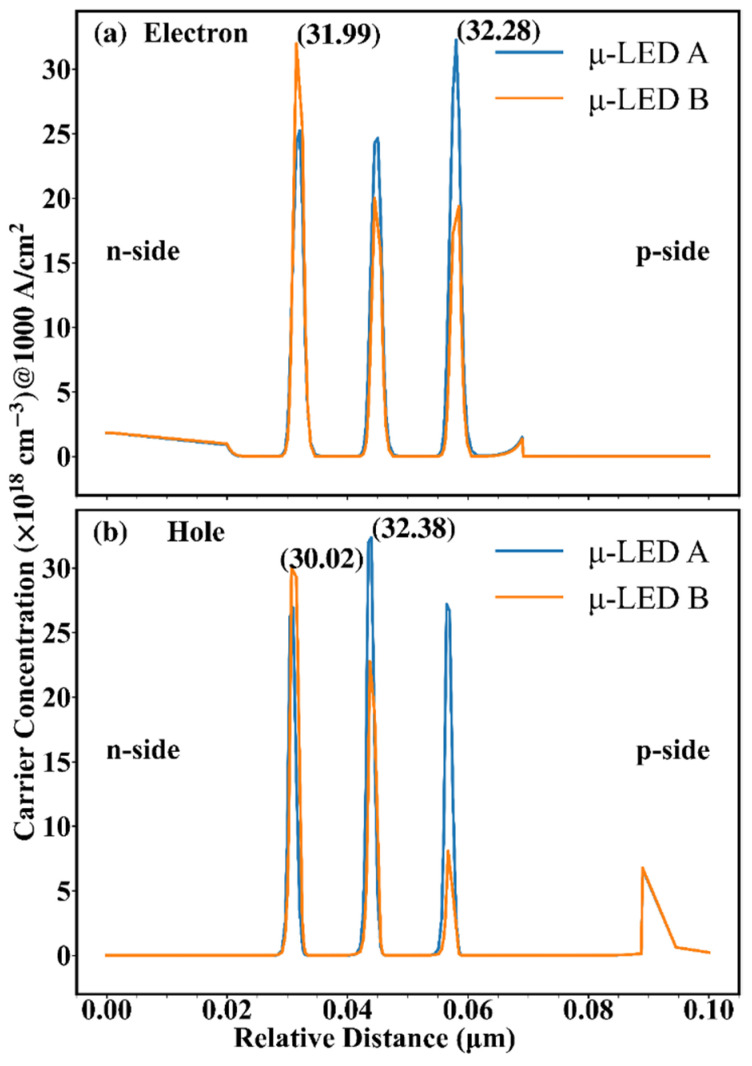
(**a**) Electron distribution and (**b**) hole distribution at 1 kA/cm^2^ for μ-LED A and μ-LED B.

To understand the internal differences between the two devices, Figure 3 shows the electron and hole concentrations as a function of vertical distance relative to the n-side (relative distance) for the two μ-LEDs at 1 kA/cm^2^. It can be seen that the electron concentration reaches a maximum near the p-side in μ-LED A, but that reaches a maximum near the n-side in μ-LED B. Besides, the hole concentration reaches a maximum at the middle in μ-LED A, but that reaches a maximum near the n-side in μ-LED B. In Figure 3, the peaks of the carrier concentration of the two μ-LEDs are selected and marked. It can be found that the peaks of the carrier concentration of μ-LED A are higher than those of μ-LED B. The above shows that the carrier distribution can be changed by adjusting the structure of QWs, and the cause of which needs to be further analyzed.

**Figure 4:**
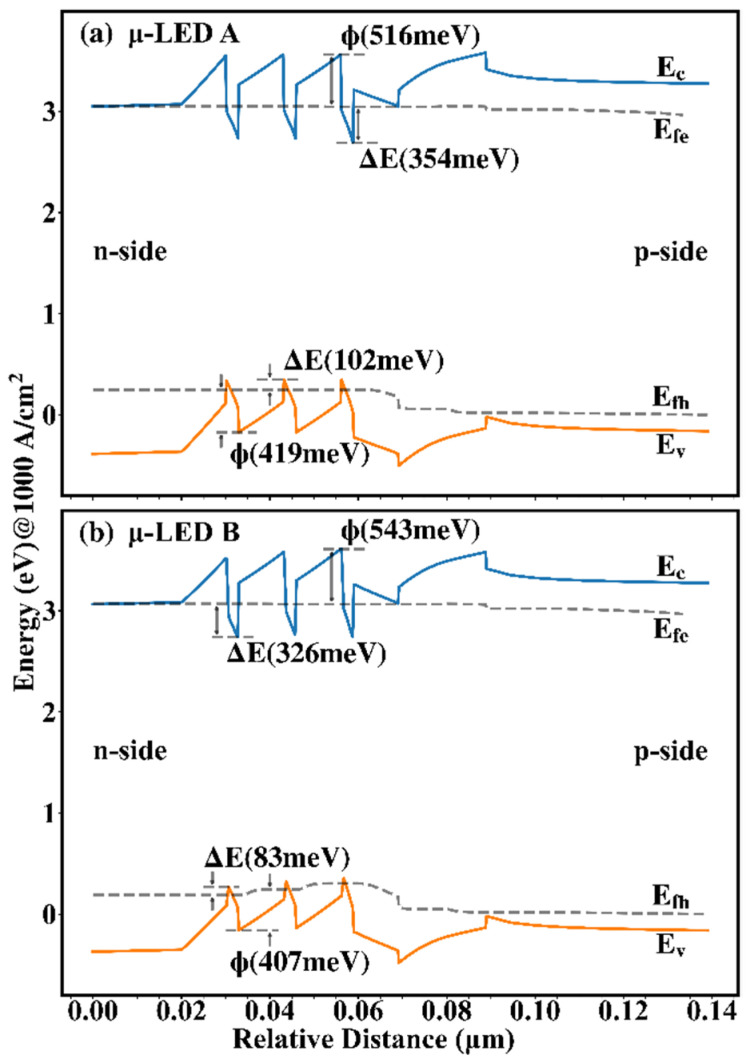
Energy band of (**a**) μ-LED A and (**b**) μ-LED B at 1 kA/cm^2^.

In order to study the variation rules of carrier concentration, Figure 4 shows the energy band as a function of relative distance for the two μ-LEDs at 1 kA/cm^2^. The Φ is used to represent the energy gap between QB and QW, calculated by the difference between the peak energy of the next QB and the energy valley of the QW, reflecting the transport capacity of carriers. The larger the Φ, the higher the barrier of the QB, the harder the carriers escape, and the weaker the carrier transport. In Figure 4, the Φs are marked at the QW located in the middle position. In the conduction band, it can be found that the Φ of μ-LED B is higher than that of μ-LED A, indicating the stronger electron transport in μ-LED A, which explains the different electron distribution between μ-LED A and μ-LED B in Figure 3. On the contrary, in the valence band, it can be found that the Φ of μ-LED A is higher than that of μ-LED B, indicating the enhanced hole transport in μ-LED B, which explains the different hole distribution between μ-LED A and μ-LED B in Figure 3. The ΔE is used to represent the energy gap in the QW, calculated by the difference between the Fermi energy level and the energy valley of the QW, reflecting the confinement ability of carriers. The larger the ΔE, the easier the carriers are trapped by the QW, and the higher the carrier concentration in the QW. In Figure 4, the ΔEs are marked at the QW with the highest carrier concentration. It can be found that the ΔEs of μ-LED A are higher than those of μ-LED B due to the less lattice mismatch in trapezoidal QWs, which explains the higher peaks of the carrier concentration of μ-LED A than those of μ-LED B in Figure 3.

**Figure 5:**
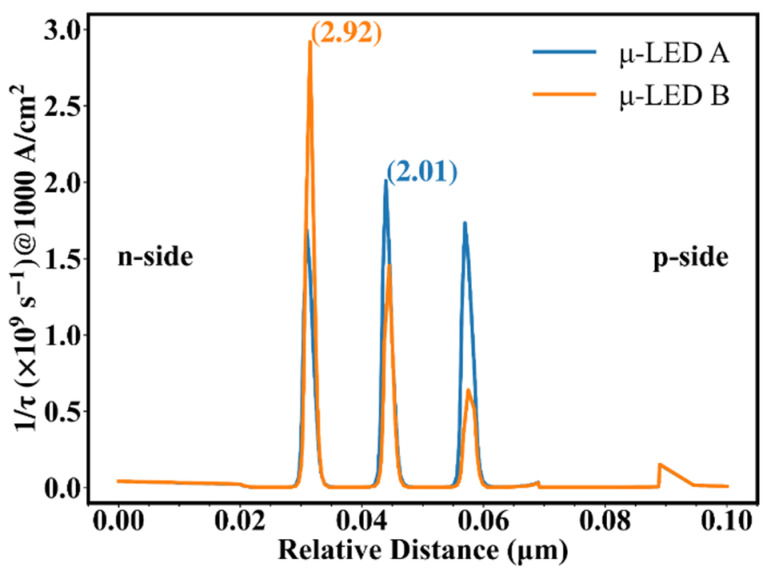
Reciprocal carrier lifetime vs. relative distance at 1 kA/cm^2^ for μ-LED A and μ-LED B.

Based on Equations (3) and (4), it can be concluded that the −3 dB modulation bandwidth is positive relative to 1/τ, which is positive relative to carrier concentration. Figure 5 shows 1/τ as a function of relative distance for the two μ-LEDs at 1 kA/cm^2^. In Equation (3), the concentrations of electrons and holes used to calculate 1/τ are taken from the data at the corresponding locations in Figure 3, which means that 1/τ is also related to the overlap of the electron-hole distribution. Different carrier concentrations lead to different carrier lifetimes in different QWs, and the short-lived carriers reflect the high-frequency portion of the frequency response, which greatly affects the modulation bandwidth. To facilitate the analysis, the peaks of 1/τ of the two μ-LEDs are selected and marked in Figure 5. Combining Figure 3 and Figure 4, it can be found that although a higher ΔE exists in μ-LED A, the improved hole transport makes the peaks of concentrations of electrons and holes of μ-LED B almost coincide. As a result, the peaks of carrier concentrations of μ-LED B and μ-LED A have little difference. Combining Figure 2, Figure 3 and Figure 5, it can be found that although the peak of the carrier concentration of μ-LED B is slightly lower than that of μ-LED A, the huge advantage in the overlap of electron-hole wave functions allows μ-LED B to create a higher peak of 1/τ.

To further illustrate the advantages of μ-LED B, we modify the QW thickness of μ-LED A to 2.5 nm, denoted as μ-LED C (Figure S1). At 1 kA/cm^2^, we calculated the carrier distribution and carrier wave function of μ-LED C (Figure S2 and Figure S3), then compared the peak electron concentration, peak hole concentration, the peak of 1/τ (Figure S4), and the electron-hole wave functions overlap of μ-LED A, μ-LED B, and μ-LED C, respectively, as shown in Table 1. In Table 1, the QWs corresponding to the carrier concentration are marked in brackets, and the QWs from the n-side to the p-side are sequentially denoted as QW1, QW2, and QW3. Compared with μ-LED A, the carrier concentration of μ-LED C is lower, but the advantage in the overlap of electron-hole wave functions gives it a larger 1/τ. Compared with μ-LED B, the electron-hole wave function overlap of μ-LED C is slightly lower, and the peak of electron concentration is slightly lower, but the peak of hole concentration is slightly higher. Importantly, the peaks of the carrier concentration of μ-LED B are concentrated in the same QW, making the peak of 1/τ greatly increased.

**Figure 6:**
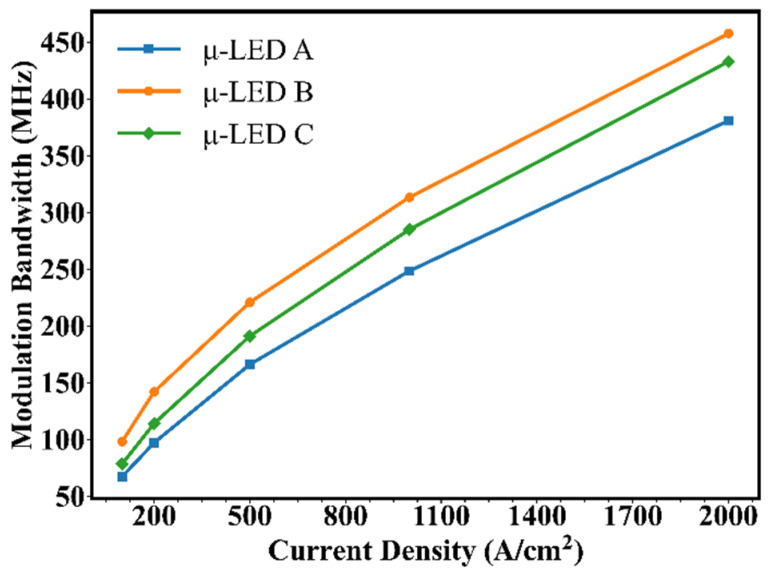
The −3 dB modulation bandwidth vs. current density for μ-LED A, μ-LED B, and μ-LED C.

Compared with μ-LED A and μ-LED C, a higher peak of 1/τ in μ-LED B can be found, implying a higher modulation bandwidth for μ-LED B. To verify our analysis, the frequency responses of the three devices are simulated. To generate a high-frequency small-amplitude signal, the current input is set as a Gaussian pulse signal with a signal width of 0.1 ns and a signal amplitude of 1% of the DC bias. In this study, the modulated input signal is converted to optical output signal by the μ-LED and then subjected to Fourier analysis to obtain the frequency response. Figure 6 shows the −3 dB modulation bandwidth vs. the current density of the three μ-LEDs. It can be seen that the −3 dB modulation bandwidth of μ-LEDs increases with increasing current density, which is attributed to the decrease in carrier lifetime. Moreover, the growth of the −3 dB modulation bandwidth of μ-LEDs gradually slows down as the current density increases, attributed to slower carrier concentration growth and more electron leakage. Furthermore, it can be found that the modulation bandwidth of μ-LED B is always higher than that of μ-LED A and μ-LED C as the current density shifts from 100 A/cm^2^ to 2 kA/cm^2^, and the modulation bandwidth can be raised to 457.5 MHz. For μ-LED B, the improved hole transport allows the peak of the concentration of holes to meet that of electrons in the same QW, and the large electron-hole wave function overlap greatly reduces the differential carrier lifetime. Actually, a μ-LED with trapezoidal QWs can maximize the peak value of 1/τ, thereby maximizing the modulation bandwidth.

**Figure 7:**
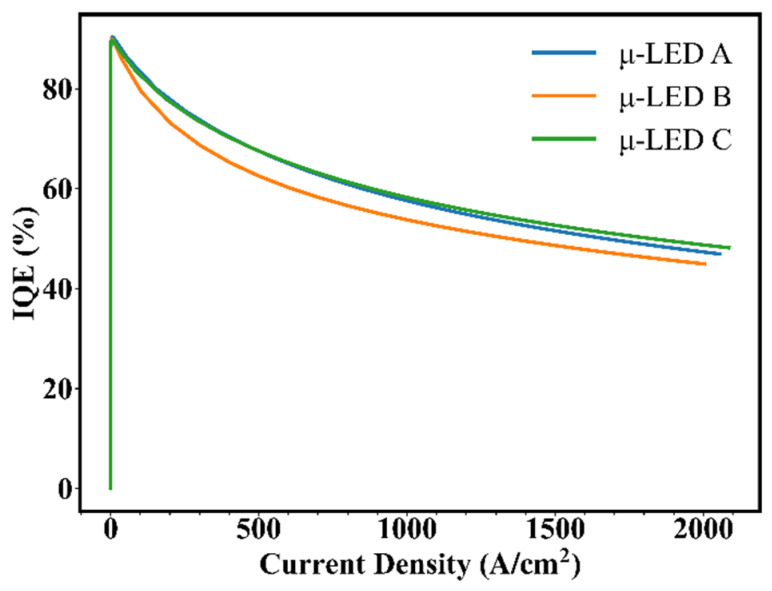
IQE vs. current density for μ-LED A, μ-LED B, and μ-LED C.

In addition to modulation bandwidth, the quantum efficiency is also an important parameter when considering VLC application scenarios. Therefore, we further compared the internal quantum efficiencies (IQEs) of μ-LED A, μ-LED B, and μ-LED C, as shown in Figure 7. It can be seen that the IQE of μ-LED B is slightly lower than that of μ-LED A and μ-LED C. Furthermore, the relationship between modulation bandwidth and IQE for the μ-LED A, μ-LED B, and μ-LED C was analyzed. At 500 A/cm^2^, the modulation bandwidths of μ-LED A, μ-LED B, and μ-LED C are 166.1 MHz, 220.8 MHz, and 191.1 MHz, respectively, while the IQEs of those are 66.9%, 62.5%, and 67.4%. Taking μ-LED A sample as a reference, the modulation bandwidth of μ-LED B is increased by about 32.9%, but the IQE is decreased by about 6.57%, while the modulation bandwidth of μ-LED C is increased by about 15.1%, and the IQE is increased by about 0.75%. Our results prove that there is a trade-off between modulation bandwidth and IQE, and we believe that it is worthwhile to optimize the design of MQWs for high-efficiency VLC devices.

## 4. Conclusions

The modulation bandwidths of μ-LEDs with different QW structures were compared, and the physical mechanisms involved were discussed in detail. Compared with μ-LEDs with 3 nm and 2.5 nm thick QWs, μ-LEDs with trapezoidal QWs are superior in both carrier distribution and electron-hole wave function overlap, exhibiting a higher modulation bandwidth. It is desired that the modulation bandwidth will continue to increase as the QW thickness continues to decrease, but the trapezoidal QW structure we designed is still excellent at lower current densities, where the μ-LED can operate more efficiently. Although it is natural to use thinner QWs, our research provides an alternative design for developing high-efficiency and high-speed μ-LEDs for VLC application.

## Figures and Tables

**Table 1:** Peak electron concentration, peak hole concentration, electron-hole wave function overlap, and peak 1/τ for μ-LED A, μ-LED B, and μ-LED C at 1 kA/cm^2^.

Physical Value at 1 kA/cm^2^	μ-LED A	μ-LED B	μ-LED C
Peak Electron Concentration (×10^18^ cm^−3^)	32.28 (QW3)	31.99 (QW1)	30.23 (QW3)
Peak Hole Concentration (×10^18^ cm^−3^)	32.38 (QW2)	30.02 (QW1)	31.20 (QW2)
Electron-Hole Wave Function Overlap	0.617	0.784	0.737
Peak 1/τ (×10^9^ s^−1^)	2.011	2.920	2.033

## Data Availability

The data that support the findings of this study are available from the corresponding authors upon reasonable request.

## Supplementary Materials

The following are available online at https://www.mdpi.com/article/10.3390/nano12213818/s1, Figure S1: Structure of μ-LED C with 2.5 nm thick quantum wells, Figure S2: Electron distribution and hole distribution at 1 kA/cm^2^ for μ-LED C, Figure S3: Overlaps of electron-hole wave function vs. current density for μ-LED A, μ-LED B, and μ-LED C, Figure S4: Reciprocal carrier lifetime vs. relative distance at 1 kA/cm^2^ for μ-LED C.
