# Supplementary material for: Study on Modulation Bandwidth of GaN-Based Micro-Light-Emitting Diodes by Adjusting Quantum Well Structure
Source: Nanomaterials (Basel). 2022 Oct 28;12(21):3818. doi: 10.3390/nano12213818 (PMC9659067; doi:10.3390/nano12213818)
Supplement: Supplementary file 1 [file nanomaterials-12-03818-s001.zip › nanomaterials-1888154-supplementary.pdf]

nanomaterials

Supplementary Material

Study on modulation bandwidth of GaN-based micro-light-emitting diodes by adjusting quantum well structure

Pan Yin[1], Ting Zhi[2], Tao Tao[3,*] and Xiaoyan Liu[1,2,*]

[1] Institute for Electric Light Sources, School of Information Science and Technology, Fudan University, Shanghai 200438, China
[2] College of Integrated Circuit Science and Engineering, and National and Local Joint Engineering Laboratory for RF Integration and Micro-Packaging Technologies, Nanjing University of Posts and Telecommunications, Nanjing 210023, China
[3] Key Laboratory of Advanced Photonic and Electronic Materials, School of Electronic Science and Engineering, Nanjing University, Nanjing 210046, China
* Authors to whom correspondence should be addressed: ttao@nju.edu.cn; xiaoyanliu@njupt.edu.cn

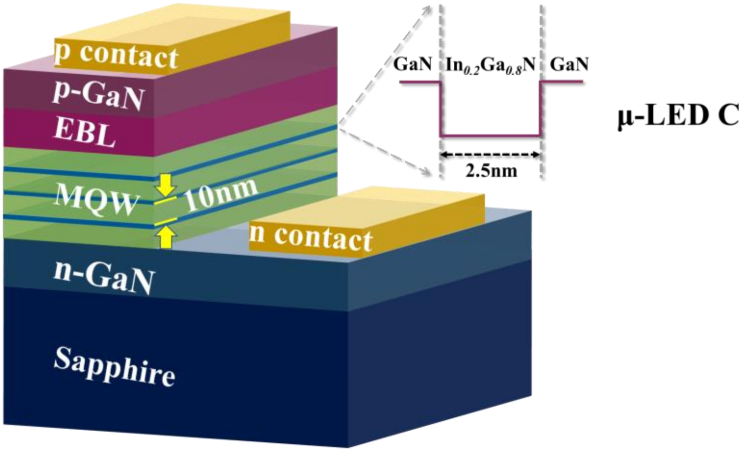

Figure S1. Structure of μ-LED C with 2.5 nm thick quantum wells.

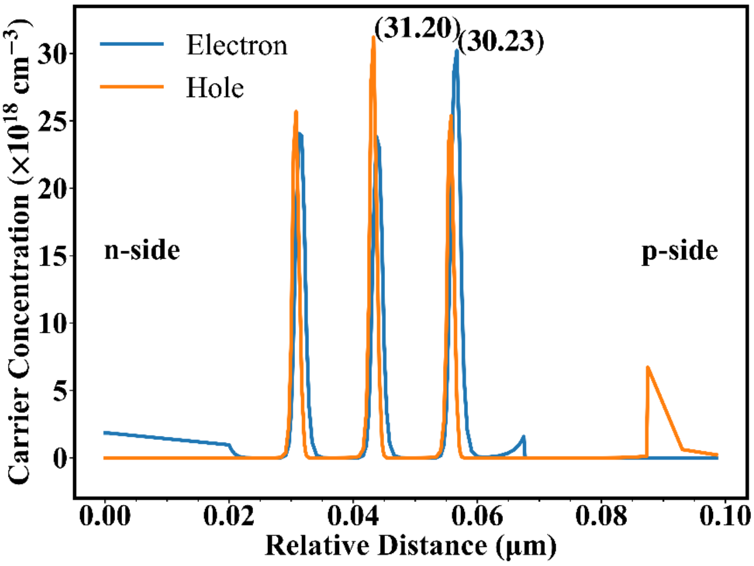

Figure S2. Electron distribution and hole distribution at 1 kA/cm² for μ-LED C.

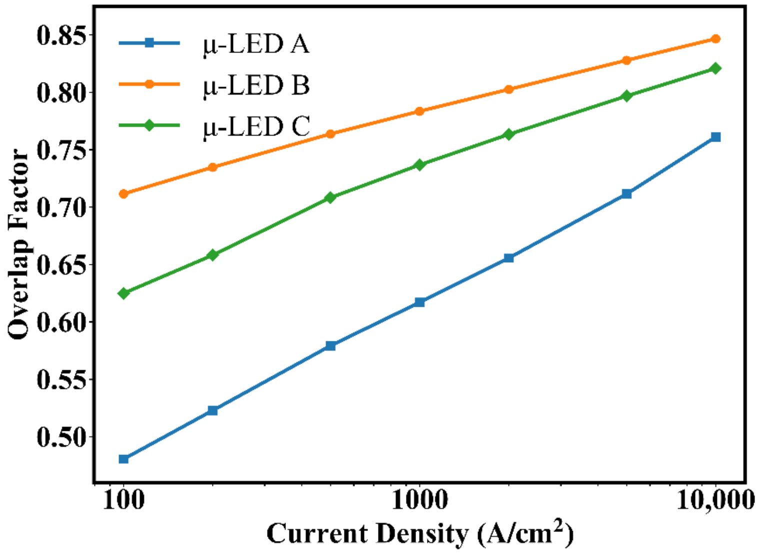

Figure S3. Overlaps of electron-hole wave function vs. current density for μ-LED A, μ-LED B, and μ-LED C.

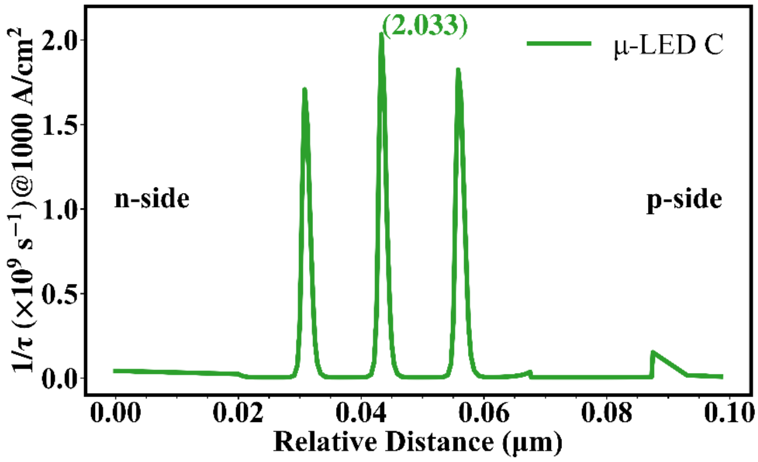

Figure S4. Reciprocal carrier lifetime vs. relative distance at 1 kA/cm² for μ-LED C.
